# Bacterial associated urinary tract infection, risk factors, and drug susceptibility profile among adult people living with HIV at Haswassa University Comprehensive Specialized Hospital, Hawassa, Southern Esthiopia

**DOI:** 10.1038/s41598-020-67840-7

**Published:** 2020-07-01

**Authors:** Netsanet Nigusse Tessema, Musa Mohammed Ali, Mengistu Hyilemeriam Zenebe

**Affiliations:** 1 Hawassa University Compressive Specialized Hospital, Awassa, Ethiopia; 2 School of Medical Laboratory Science, Hawassa University College of Medicine and Health Sciences, Awassa, Ethiopia

**Keywords:** Microbiology, Diseases, Medical research

## Abstract

People living with human immunodeficiency virus (HIV) are more likely to develop urinary tract infections (UTI) due to the suppression of their immunity. The aim of this study was to determine the prevalence, risk factors of UTI, and drug susceptibility pattern of bacteria isolated among peoples infected with HIV. A hospital-based cross-sectional study was conducted among 224 HIV positive individuals attending Hawassa University Comprehensive Specialized Hospital (HUCSH) from September 17 to November 16, 2018. Midstream urine was collected from all study participants and inoculated on to Blood and MacConkey agar. Bacterial isolates were characterized by Gram stain and standard biochemical tests. Kirby-Bauer method was used for antimicrobial susceptibility testing. Sociodemographic and clinical data were collected by a semi-structured questionnaire. Data were analyzed using SPSS version 20. A bivariate and a multivariable regression model were employed to determine the association between dependent and independent variables. From the total 224 study participants, 23 (10.3%) (95% CI 6.7–14.7) had culture-confirmed UTIs. The distributions of the bacteria were as follows: *Escherichia coli* 16 (69.6%), *Staphylococcus aureus* 2 (8.7%), *Klebsiella pneumoniae* 2 (8.7%), *Enterobacter aerogenes* 2 (8.7%) and *Pseudomonas species* 1 (4.3%). UTI prevalence was also high among study participants with a previous history of UTI and CD4^+^ count < 200/mm^3^. Female study participants were about five times more likely to have UTI (AOR 5.3, 95% CI 1.5–19.2). Ninety-three percent of bacteria isolated were susceptible to nitrofurantoin, ceftriaxone, and gentamycin; 87.5% were susceptible to meropenem and norfloxacin; whereas 93.8%, 68.8%, and 62.5% of isolates were resistant to ampicillin, tetracycline, and cotrimoxazole respectively. Multidrug resistance (MDR) was seen in 18 (78.3%) of bacterial isolates.

## Introduction

Urinary tract infection (UTI) is an important health problem that can affect HIV positive individuals. UTI is defined as the presence of significant number of bacteria (greater than or equal to 10^5^) in the urine ^1^. Individuals who are infected with Human Immunodeficiency Virus (HIV) are at increased risk of acquiring UTI because of several reasons, mainly due to suppression of immune system^2^. UTI can be asymptomatic and asymptomatic. Among HIV infected individuals, asymptomatic UTI can progress to symptomatic UTI characterized by mild irritation during voiding to bacteremia, sepsis, and death^3–5^.

Urinary tract infections are mostly caused by bacteria and it may progress to blood infection and pyelonephritis in individual with some underlying risks^6^. UTI infection may leads to hospitalization of HIV infected patients^7^. HIV infected population are disproportionately affected by UTI as result of immune suppression^5,8^. Several bacteria and some fungus cause UTI among HIV-infected persons^9,10^. However, most infections are caused by enteric pathogens such as *Escherichia coli*^4^. There are few studies from Ethiopia: Jimma^11^, Gondar^12^, Harar^13^, and two from Addis Ababa^14,15^ that reported UTI among HIV patients.

Urinary tract infection is a serious public healthcare problem that causes a high economic burden to the country and decreases the quality of life^5,16^. Worldwide there are about 150 million individuals who are diagnosed with UTI^17^. UTI affects HIV infected population considerably^18,19^. HIV positive individuals, whose CD4 cell is less than 500 cells/mm^3^ are commonly affected by UTI^15^. According to study from Tanzania, a CD4^+^ cell count less than 200 per microliter was significantly associated with UTI among HIV positive individuals^20^. A report from Europe also indicated HIV infected individuals with a CD4^+^ cell count less than 200/mm^3^ are more liklely to experience UTI^21^. UTI commonly affect femalses than males among general population and also among HIV infected population^22^. Management of other non-HIV associated diseases including UTI in people living with HIV has become increasingly important. Additionally, it is complicated by emergence of antibiotic resistant bacteria making treatment of UTI challenging. In these regard, UTI co-infection with HIV is becoming a major challenge and leads to additional costs^8,18^.

There are scarce data regarding the causative agents of UTI among HIV positive individuals from the study area; therefore, this study was conducted to determine the prevalence of bacterial UTI, risk factors and drug susceptibility pattern of isolates among adult people living with HIV attending HUCSH, Hawassa Ethiopia.

## Methods

### Study area

Hawassa is the capital city of the south region and located 275 km from Addis Ababa, the capital city of Ethiopia. Hawassa University Compressive Specialized Hospital was established in November 2005 and it serves about 12 million peoples. Patients seeking medical care receive services at different outpatient and inpatient units (surgery, gynecology and obstetrics, internal medicine, pediatrics, ophthalmology, psychiatry, radiology, pathology). The hospital also provides different services for individuals living with HIV in the ART clinic. The total number of HIV infected individuals attending HUCSH was 2,856. In government hospital of Ethiopia including the study area, HIV infection is diagnosed by using repaid serological tests. The drugs used to the treatment of HIV infected individuals are: Zidovudine, Lamivudine, Abacavir,Efavireniz, Nevirapine, Lopinavir/ritonavir,TDF(Tenofovir Disoproxil fumarate), Doltugravir, Ataznavir. Previously the treatment was initiated when CD4^+^ cell count is less than 350/mm^3^ but currently it the treatment is given based on viral load (> 1,000 copies/µl).

### Study design and period

A Hospital-based cross-sectional study was conducted from September 17 to November 16, 2018.

### Source population

All adult people living with HIV and those who attended HUCSH, ART clinic during the study period for follow up.

### Study population

HIV positive adults (both symptomatic and asymptomatic for UTI) were selected by systematic random sampling method were the study population.

### Eligibility

Individual aged greater or equal to 18 years and HIV positive were included in the study. Individuals on antibiotic therapy for the last 2 weeks prior to data collection were excluded.

### Variables of the study

Dependent variables include the prevalence of UTI, drug susceptibility pattern; Independent variables include age, Sex, marital status, occupational status, educational level, clinical signs and symptoms of UTI, previous history of UTI, previous history of catheterization, cotrimoxazole usage, CD4^+^ cell count.

### Sample size determination

The required sample size was determined by using single population formula considering the following assumptions: A prevalence of 15.8% from the previous study conducted in Ethiopia^14^ and margin of error (d) 5%, 95 confidence interval, and 10% non-response rate. Based on the above assumption the total sample size was 224.

### Operational definition

Urinary tract infection: is the presence of pathogenic microorganisms within the urinary tract in a significant quantity (≥ 10^5^ cfu/ml)^23^.

Multi-drug resistance bacteria: are bacteria resistant for greater than two different classes of drug categories^24^.

### Sampling technique

To recruit study participants we used a systematic random sampling method. We followed patient flow at ART clinic for one week. The average patient flow per day was 22 and the data collection period was 2 months. By dividing the sample size (N = 224) for the data collection period (48 days) we arrived at the sample size that could be collected per day, which is equal to 5. K value was calculated by dividing the average number of participants per day (n = 22) to participants recruited by day (n = 5), K was 4. By using a lottery method one participant was selected from the 1st 4 attendants then systematically every 4 participants were selected until the required sample size was obtained.

### Data collection

Sociodemographic, associated factors and clinical data were collected by attending nurses using semi-structured questionnaire. The participants’ current CD4^+^ cell value was taken from their medical records (It was performed by using BD FACSPresto).

### Sample collection

A urine sample was collected after adequate explanation/information was provided by attending laboratory professionals. Participants were instructed to collect about 30 ml of midstream urine (MSU) for microbiological examination by giving a sterile, dry, wide-necked, leak-proof container. Urine samples were processed immediately at HUCSH microbiology laboratory. If there were a delay the samples were stored in the refrigerator at 2–8 °C.

### Urine culture and biochemical test

Using a calibrated loop 0.001 ml of well-mixed un-centrifuged urine was inoculated on blood and MacConkey agar. The inoculated media were incubated at 35–37 °C for 24 h and examined for the growth of bacteria^25^. For identification of Gram negative bacteria, bacterial colony was sub-cultured onto nutrient broth. Then, the nutrient broth was inoculated on biochemical test culture medias such as Triple sugar iron agar, Simmon’s citrate agar, Lysine iron agar, Urea, Motility tests, and Indol. Identification of species was done by their characteristics in the respective culture media as per the standard. Species identification for Gram positive bacteria was carried out using catalase and coagulase test^25^.

### Antimicrobial susceptibility testing

Drug susceptibility testing was done by following the Kirby-Bauer disk diffusion method on Mueller–Hinton agar (Oxoid Ltd, Hampshire, UK)^26^. The tested drugs includes ampicillin (10 µg), ciprofloxacin (5 µg), cotrimoxazole (23.75 µg), gentamicin (10 µg), meropenem (10 µg), nitrofurantoin (300 µg), augumentin (20 µg), ceftriaxone (30 µg), norfloxacin (10 µg), ceftazidime (30 µg), tetracycline (30 µg), clindamycin (2 µg), penicillin (10 µg), erythromycin (15 µg), and cefoxitin (30 µg). Briefly, using a sterile wire loop, 3–5 pure similar colonies were mixed in 5 ml of normal saline until the turbidity of the suspension matches 0.5 McFarland standards. By using a dry, sterile cotton swab, a portion of suspension was inoculated on the surface of the Mueller Hinton agar plate (MHA). Selected antibiotics disks were placed on MHA by using forceps and incubated at 37 °C for 18–24 h. The zone of inhibition (diameter) around the antibiotic disk was measured; bacteria were classified as susceptible, intermediate and resistant according to Clinical Laboratory Standard Institute (CLSI) guidelines 2019^26^.

### Data quality assurance

To ensure the quality of sociodemographic and clinical data, the semi-structured questionnaire was pretested and data collectors were trained. Sterility of culture media was checked by incubating 5% of culture media overnight at 35–37 °C without specimen inoculation. The performance of culture media was checked by suing control strains. Any physical changes like cracks, excess moisture, color, hemolysis, dehydration, and contamination was assessed and expiration date was also checked. Standard strains of *E. coli* (ATCC 25922) and *S. aureus* (ATCC 25923) were used as quality control throughout the study for culture and antimicrobial susceptibility tests.

### Data processing and analysis

The data was analyzed using SPSS version 20. The bivariate regression model was employed to examine the associations between dependent and independent variables. Based on the bivariate analysis a variables with *P* value ≤ 0.25 were selected for further analysis using a multivariable regression model. A *P* value < 0.05 was considered as statistically significant and results were presented by using odds ratio and 95% level of confidence.

## Results

### Sociodemographic characteristics

In the current study, a total of 224 people with HIV participated with zero no response rates. The age of the participant ranged from 18 to 59 years with a mean and median age of 39 years. The majority of the study participants were females 131 (58.5%). Most of the participants belong to the age category 28–37 years. The majority of study participants completed elementary school (Table 1).

**Table 1:** Sociodemographic characteristics of people living with HIV who were attending Hawassa University Comprehensive Specialized Hospital ART clinic, Hawassa, Ethiopia, from September 17 to November 16, 2018 (n = 224).

Characteristics	Frequency	Percent (%)
Sex		
Male	93	41.5
Female	131	58.5
Age		
18–27	15	6.7
28–37	85	37.9
38–47	77	34.4
48–57	39	17.4
≥ 58	8	3.6
Residence		
Urban	218	97.3
Rural	6	2.7
Marital status		
Married	103	46
Single	43	19.2
Divorced	45	20.1
Widowed	33	14.7
Occupational status		
Employee	52	23.2
Daily laborer	42	18.8
Merchant	66	29.5
Housewives	53	23.7
Others^a^	11	4.9
Educational status		
University graduate	31	13.8
High school	60	26.8
Elementary	96	42.9
Illiterate	37	16.5

^a^Others: driver, student and farmer.

### Prevalence of Urinary tract infection among HIV positive individuals

Out of the 224 study participants, bacteria were isolated from 23 giving an overall prevalence of 10.3% (95% CI 6.7–14.7) UTI. Symptomatic and asymptomatic UTI was 11 (4.9%) and 12 (5.4%) respectively. Twenty-one (91.3%) of isolates were Gram-negative bacteria. *E. coli* was the most predominant isolates. The proportion of bacteria isolated is as follows: 16 (69.6%) *E. coli,* 2 (8.7%) *K. pneumoniae,* 2 (8.7%) *E. aerogenes*, 2 (8.7%) *S. aureus* and 1 (4.3%) *Pseudomonas species.*

### Factors associated with the prevalence of UTI among HIV positive individuals

Based on bivariate regression analysis variables with *P* value ≤ 0.25 (sex, age and marital status) were selected for further multivariable regression analysis and female study participants showed at least five times more likely to have significant bacteriuria (AOR 5.3; 95% CI 1.5, 19.2) when compared to male study participants (*P* = 0.012). However, there was no statistically significant association with age and marital status (*P* > 0.05) (Table 2).

**Table 2:** Bivariate and multivariate analysis of sociodemographic characteristics and UTI among people living with HIV who were attending Hawassa University Comprehensive Specialized Hospital ART clinic, Hawassa, Ethiopia, from September 17 to November 16, 2018 (n = 224).

Variables	UTI		COR (95% CI)	*P* value	AOR (95% CI)	*P* value
	Yes, n (%)	No, n (%)				
Sex						
Male	3 (3.2)	90 (96.8)	1	0.024	1	0.012
Female	20 (15.3)	111 (84.7)	5.5 (1.3, 21.1)		5.3 (1.5, 19.2)	
Age						
18–27	3 (20)	12 (80)	1			
28–37	6 (7.1)	79 (92.9)	0.3 (0.1, 1.4)	0.123		
38–47	6 (7.8)	71 (92.2)	0.3 (0.1, 1.5)	0.161		
48–57	7 (17.9)	32 (82.1)	0.9 (0.2, 3.4)	0.862		
≥ 58	1 (12.5)	7 (87.5)	0.6 (0.1, 6.6)	0.654		
Residence						
Urban	22 (10.1)	196 (89.9)	1	0.605		
Rural	1 (16.7)	5 (83.3)	1.8 (0.2, 15.9)			
Educational status						
University graduate	2 (6.5)	29 (93.5)	1			
High school	5 (8.3)	55 (91.7)	1.3 (0.2, 7.2)	0.750		
Elementary	13 (13.5)	83 (86.5)	2.3 (0.5, 10.7)	0.299		
No formal education	3 (8.1)	34 (91.9)	1.3 (0.2, 8.2)	0.795		
Marital status						
Married	10 (9.7)	93 (90.3)	1			
Single	2 (4.7)	41 (95.3)	0.5 (0.2.2)	0.321		
Divorced	5 (11.1)	40 (88.9)	1.2 (0.4, 3.6)	0.795		
Widowed	6 (18.2)	27 (81.8)	2.1 (0.7, 6.2)	0.195		
Occupation						
Government employee	6 (11.5)	46 (88.5)	1			
Daily laborer	5 (11.9)	37 (88.1)	1.0 (0.3, 3.7)	0.956		
Merchant	6 (9.1)	60 (90.9)	0.8 (0.2, 2.5)	0.663		
House wife	6 (11.3)	47 (88.7)	0.9 (0.3, 3.3)	0.972		
Others^a^	–	11 (100)	0.0 (0.0, 0.0)	0.999		

*UTI* urinary tract infection, *COR* crude odd ration, *AOR* adjusted odd ratio, *CI* confidence interval, *n* number.

^a^Others: driver, student and farmer.

Based on bivariate logistic analysis variables with *P* value ≤ 0.25 such as fever, diabetics, previous history of UTI, previous history of catheterization and CD4 count were further selected for multivariable logistic analysis. Previous history of UTI (AOR 4.4; 95% CI: 1.6, 11.7) and CD4 count less than 200 (AOR 4.9; 95% CI 1.2, 18.5) were significantly associated with UTI. However, there was no statistically significant association between UTI and fever, diabetes and previous history of catheterization (*P* > 0.05) (Table 3).

**Table 3:** Bivariate and multivariate analysis of clinical characteristics and UTI among people living with HIV who were attending Hawassa University Comprehensive Specialized Hospital ART clinic, Hawassa, Ethiopia, from September 17 to November 16,2018 (n = 224).

Variables	UTI		COR (95% CI)	*P* value	AOR (95% CI)	*P* value
	Yes, n (%)	No, n (%)				
Fever						
No	18 (8.6)	191 (91.4)	1	0.005		
Yes	5 (33.3)	10 (66.7)	5.3 (1.6, 17.2)			
Dysuria						
No	22 (10.7)	183 (89.3)	1	0.463		
Yes	1 (5.3)	18 (94.7)	0.5 (0.1, 3.6)			
Diabetics						
No	20 (9.5)	191 (90.5)	1	0.132		
Yes	3 (23.1)	10 (76.9)	2.9 (0.7, 11.3)			
Cotrimoxazole usage as prophylaxis						
Yes	5 (12.5)	35 (87.5)	1	0.609		
No	18 (9.8)	166 (90.2)	0.8 (0.3, 2.2)			
Frequency of urination						
No	21 (10.8)	174 (89.2)	1			
Yes	2 (6.9)	27 (93.1)	0.6 (1.2, 2.8)	0.525		
Urgency of urination						
No	22 (10.8)	181 (89.2)	1			
Yes	1 (4.8)	20 (95.2)	0.4 (0.5, 3.2)	0.397		
Flank pain						
No	21 (1.2)	166 (88.8)	1			
Yes	2 (5.4)	35 (94.60	0.5 (0.1, 2.0)	0.298		
Renal stone						
No	22 (10.0)	197 (90)	1			
Yes	1 (20)	4 (80)	2.2 (0.2, 20.9)	0.480		
Previous history of UTI						
No	14 (7.7)	169 (92.3)	1		1	0.004
Yes	9 (22)	32 (78)	0.3 (0.1, 0.7)	0.009	4.4 (1.6, 11.7)	
History of catheterization						
No	20 (9.20)	198 (90.8)	1			
Yes	3 (50)	3 (50)	9.9 (1.9, 52.3)	0.007		
CD4 count						
≥ 200	17 (8.30)	188 (91.7)	1		1	0.017
< 200	6 (31.6)	13 (68.4)	5.1 (1.7, 15.1)	0.003	4.9 (1.2, 18.5)	

*AOR* adjusted odds ratio, *COR* crude odd ration, *AOR* adjusted odd ratio, *CI* confidence interval, *n* number.

### Antimicrobial susceptibility profile

From the total of *E. coli* (n = 16) isolated in this study, 15 (93.8%), 11 (68.8%), 10 (62.5%) were resistant to ampicillin, tetracycline and to cotrimoxazole respectively. 16 (100%) of *E. coli* were susceptible to nitrofurantoin and ceftriaxone (Table 4).

**Table 4:** Antimicrobial susceptibility pattern of Gram-negative and Gram positive bacteria isolated from urine culture of people living with HIV attending Hawassa University Comprehensive Specialized Hospital ART clinic, Hawassa, Ethiopia, from September 17 to November 16, 2018 (N = 23).

Bacterial isolates (n)	Pattern	AMP, n (%) n	CIP, n (%)	COT n (%)	GN, n (%)	MER n (%)	NIT, n (%)	AUG, n (%)	CTR, n (%)	NOR, n (%)	CAZ, n (%)	TET, n (%)	CLN, n (%)	PEN, n (%)	E, n (%)	CFT, n (%)
*E. coli* (16)	S	–	14 (87.5)	6 (37.5)	15 (93.8)	14 (87.5)	16 (100)	11 (68.8)	16 (100)	14 (87.5)	8 (50)	5 (31.3)	NA	NA	NA	NA
	I	1 (6.3)	1 (6.3)	-	–	–	–	–	–	1 (6.3)		–	NA	NA	NA	NA
	R	15 (93.8)	1 (6.3)	10 (62.5)	1 (6.3)	2 (12.5)	–	5 (31.3)	–	1 (6.3)	8 (50)	11 (68.8)	NA	NA	NA	NA
*K. pneumoniae* (2)	S	–	2 (100)	2 (100)	2 (100)	2 (100)	2 (100)	–	1 (50)	2 (100)	2 (100)	1 (50)	NA	NA	NA	NA
	I	–	–	–	–	–	–	–	–	–		1 (50)	NA	NA	NA	NA
	R	2 (100)	–	–	–	–	–	2 (100)	1 (50)	–	–	–	NA	NA	NA	NA
*E. aurogene* (2)	S	–	2 (100)	1 (50)	2 (100)	2 (100)	2 (100)	1 (50)	1 (50)	2 (100)	1 (50)	1 (50)	NA	NA	NA	NA
	I	–	–	–	–	–	–	1 (50) –	1 (50)	–	1 (50)	–	NA	NA	NA	NA
	R	2 (100)	–	1 (50)	–	–	–		–	–		1 (50)	NA	NA	NA	NA
*Pseudomonas spp.* (1)	S	–	1 (100)	–	1 (100)	1 (100)	–	1 (100)	1 (100)	–	1 (100)	–	NA	NA	NA	NA
	I	–	–	–	–	–	–	–	–	–		–	NA	NA	NA	NA
	R	1 (100)	–	1 (100)	–	–	1 (100)	–	–	1 (100)		1 (100)	NA	NA	NA	NA
*S. aureus (2)*	S	NA	2 (100)	–	2 (100)	NA	2 (100)	NA	NA	2 (100)	NA	–	2 (100)	2 (100)	2 (100)	2 (100)
	I	NA	–	–	–	NA	–	NA	NA	–	NA	–	–	–	–	–
	R	NA	–	2 (100)	–	NA	–	NA	NA	–	NA	2 (100)	–	–	–	–

*NA* not applicable, *S* suseptible, *I* intermediate, *R* resistant, *AMP* ampicillin, *CIP* ciprofloxacin, *MER* meropenem, *CAZ* ceftazidime, *CTR* ceftriaxone, *NIT* nitrofurantoin, *AUG* augumentin, *GN* gentamicin, *NOR* norfloxacin, *TET* tetracycline, *COT* cotrimoxazole, *PEN* penicillin, *E* erythromycin, *CLN* clindamycin, *CFT* cefoxitin.

### Multi-drug resistance pattern of isolated bacteria

None of the isolates was susceptible or resistant to all the drugs in the testing panel. Among the total isolates (n = 23), 18 (78.3%) were MDR. From 21 g negative bacterial isolates 16 (76.2%) showed MDR of these, 12 (57.1%) *E. coli* were MDR (Table 5).

**Table 5:** Multi-drug resistance pattern of bacterial isolates from urine culture of adult people living with HIV attending Hawassa University Comprehensive Specialized Hospital ART clinic, Hawassa, Ethiopia, from September 17–November 16, 2018 (N = 23).

Bacterial isolate	Total *n *(%)	R1	R2	R3	R4	R5	R6	MDR *n* (%)
Gram negative	21 (91.3)	1 (6.3)	4 (25)	6 (37.5)	2 (12.5)	6 (37.5)	2 (12.5)	16 (76.2)
*E. coli*	16 (76.2)	1	3	5	1	5	1	12 (57.1)
*K. pneumoniae*	2 (9.5)	–	–	1	1	–	–	2 (9.5)
*E. aerogenes*	2 (9.5)	–	1	–	–	–	1	1 (4.8)
*Pseudomonas spp.*	1 (4.8)	–	–	–	–	1	–	1 (4.8)
Gram positive *S. aureus*	2 (8.7)	–	–	2 (100)	–	–	–	2 (100)
	2 (100)	–	–	2	–	–	–	2 (100)
Total	23 (100)	1 (4.3)	4 (17.4)	8 (34.8)	2 (8.7)	6 (26.1)	2 (8.7)	18 (78.3)

*R* resistance, *n* number, *MDR* resistance for three group of drug.

R1—resistance to one group of drug; R2—resistance to two group of drug; R3—resistance to three group of drug; R4—resistance to four group of drug; R5—resistance to five group of drug; R6—resistance to six group of drug.

## Discussion

The overall prevalence of UTI among HIV positive individuals in the current study was 10.3%. The result of this finding is consistent with other studies carried out in Gondar, Ethiopia (10.7%)^12^ and Jimma, Ethiopia (12%)^11^. While a high prevalence of UTI was recorded from India (77.5%)^27^, South Africa (48.7%)^19^, Warsaw (23.2%)^5^, and Nigeria (21.1%)^6^. On the other hand, a low prevalence of 5.8% was recorded from Jos metropolis, Nigeria^10^. This difference may be due to the difference in sample size, the degree of the immune status of the study participants, ART use and geographical variation. Even if CD4 cell count rise as a result of initiation of HIV treatment other factors such as old age, other chronic disease can increase the risk of UTI.

In this study, HIV infected females had about 5 times the chance of developing UTIs compared to HIV infected males (*P* = 0.012). The finding of this study is in line with reports from other parts of Ethiopia. A study from Jimma, Ethiopia reported a high prevalence of UTI among females than males HIV positive individuals^11^. According to a study from Addis Ababa, Ethiopia HIV infected female study participants were three times more likely to have significant bacteriuria^14^. Additionally, a study from Gondar found a high prevalence of UTI among females^12^. Evidence from various epidemiological studies showed that UTIs were more common in females than in males^2,28^. High prevalence of UTI among female participants may be due to females have shorter and wider urethra, lack of prostatic fluid, and having moist urethra. Additionally, mechanical introduction of pathogens into the bladder and trauma increase the risk of UTI among females irrespective of their HIV serostatus^3,29^. But the finding of the current study is not comparable to the study conducted in Nigeria that reported a high prevalence of UTI in males than females^30^.

In the current study, participants with the previous history of UTI were about 4 times more likely to develop UTI (*P* = 0.004). This finding agrees with the studies conducted in Gondar, Ethiopia^12^ and Addis Ababa, Ethiopia^14^. This might be due to the presence of resistant strains from those who had the previous history of UTI.

Urinary tract infections appear to be multifactorial in patients with HIV infections as CD4^+^ level declines^13^. In the current study, the distribution of UTI according to CD4^+^ count showed that study participants with CD4^+^ count < 200/mm^3^ had a chance of 4.9 times to develop UTI (*P* = 0.017).This finding was supported by studies conducted in Ethiopia^13,15^, Nigeria^16^ and India^30^. These results imply that as CD4^+^ value declines the risk of UTI increases. There was no significant association between age, residence, educational status, occupation, and marital status with UTI in this study (*P* > 0.05).

In the current study, 91.3% of UTI was caused by Gram negative bacteria. We have noted that non-typical bacteria among UTI in our study. The predominant bacterium isolated in the current study was *E. coli* (69.6%). A similar *E. coli* predominance was reported from Jimma, Ethiopia (54.3%)^11^ and Gondar, Ethiopia (56.1%)^12^. *E. coli* predominance may be due to *E. coli* is the most common microorganism in the vaginal and rectal area^31^. In contrast, this study was inconsistent with the finding reported in Ebony State, Nigeria in which the predominant isolates were *S. aureus* (45.33%)^4^, Cape Coast, Ghana the predominant isolates were *S. aureus* (40%) and *S. saprophyticus* (21.8%)^2^. Most of the isolates reported from Tamil Nadu, India were *P. aeruginosa* (41.9%)^27^. Our finding of *K. pneumoniae* (8.7%), *S. aureus* (8.7%) and *Pseudomonas* species is greater than report from Warsaw^5^. The variation in the type of bacterial isolate may be due to sample collection technique and personal and environmental hygiene, and underlying conditions^13^.

In the present study, 80% of Gram negative bacteria were susceptible to ciprofloxacin, gentamycin, nitrofurantoin, and norfloxacin. The finding of this study is similar to findings reported from other areas^4,12,14^. Whereas 95.2% Gram negative bacteria in this study showed resistance to ampicillin and 57.1% of them were resistant to cotrimoxazole and 69.9% were resistant to tetracycline. Among Gram-negatives isolates, 93.8% of *E. coli* demonstrated resistance to ampicillin followed by tetracycline (68.8%) and co-trimoxazole (62.5%). Whereas, all isolates of *E. coli* were susceptible to ceftriaxone and nitrofurantoin followed by gentamicin (93.8%), ciprofloxacin and norfloxacin (87.5%) each and augumentin (68.8%). All *K. pneumoniae* were resistant to ampicillin and augumentin and all of them were susceptible to co-trimoxazole, gentamycin, ceftazidime, nitrofurantoin, and norfloxacin. All *S. aureus* isolated in the current study were susceptible to ciprofloxacin, norfloxacin, gentamycine, penicillin, whereas, all of them were resistant to co-trimoxazole and tetracycline.

Generally, ciprofloxacin, ceftriaxone, gentamicin, nitrofurantoin, and norfloxacin were most effective for bacteria isolated in the current study while ampicillin, cotrimoxazole and tetracycline were less effective. This agrees with the finding from Ethiopia^14^ and South Africa^19^.

In this study, MDR was observed among 78.3% of the isolated bacteria. This was higher compared to the finding reported in Mysore*,* India (58.3%)^7^ and Bangalre, India (48.55%)^16^. But it was lower than a report from Gondar, Ethiopia (95%)^12^ and Portharcourt, Nigeria (92.8%)^18^. The high prevalence of MDR seen to commonly prescribed antibiotics in this study might be due to the easy availability of drugs in the community, and inappropriate use of antimicrobial agents.

## Conclusion

In the current study, the overall prevalence of UTI among people living with HIV was 10.3%. Factors such as sex, CD4^+^ count < 200/mm^3^, and previous history of UTI were significantly associated with the prevalence of UTI. The isolated bacteria were *E. coli, K. pneumoniae, S. aureus, E. aurogenes,* and *Pseudomonas spp. E. coli* was the predominant bacteria. Most of the bacterial isolates were susceptible to ciprofloxacin, ceftriaxone, gentamicin, nitrofurantoin, and norfloxacin. Most of the isolates were resistant to ampicillin, tetracycline and co-trimoxazole. Multi-drug resistant bacteria were common in the current study. As susceptibility of bacteria to various antibiotics vary, management of UTI among HIV infected individuals should be guided by antimicrobial susceptibility testing.

### Ethics approval and consent to participate

Ethical clearance was obtained from the institutional review board (IRB) of Hawassa University College of medicine and health science. Then support letter were obtained from the hospital administration. Written informed consents were obtained from each study participants. All methods were carried out in accordance with relevant guidelines and regulation as mentioned by Declaration of Helsinki.

## Data Availability

All relevant data are available within the paper.

## Abbreviations

PDR: Proliferative diabetic retinopathy
DME: Diabetic macular edema
ADED: Advanced Diabetic Eye Disease
LMIC: Low and middle-income countries
PRP: Panretinal photocoagulation
NVE: Retinal neovascularization
AIDS: Acquired immune deficiency syndrome
ART: Antiretroviral therapy
ASB: Asymptomatic bacteriuria
HIV: Human immunodeficiency virus
UTI: Urinary tract infection
HUCSH: Hawassa University Comprehensive Specialized Hospital
MDR: Multi drug-resistant
